# Analysing Edge Computing Devices for the Deployment of Embedded AI

**DOI:** 10.3390/s23239495

**Published:** 2023-11-29

**Authors:** Asier Garcia-Perez, Raúl Miñón, Ana I. Torre-Bastida, Ekaitz Zulueta-Guerrero

**Affiliations:** 1 Digital, TECNALIA, Basque Research and Technology Alliance (BRTA), Parque Tecnológico de Álava Albert Einstein 28, 01510 Vitoria-Gasteiz, Álava, Spain; raul.minon@tecnalia.com; 2 Digital, TECNALIA, Basque Research and Technology Alliance (BRTA), Astondo Bidea, Edificio 700, 48160 Derio, Biscay, Spain; 3 System Engineering and Automation Control Department, University of the Basque Country (UPV/EHU), Nieves Cano, 12, 01006 Vitoria-Gasteiz, Álava, Spain; ekaitz.zulueta@ehu.eus

**Keywords:** edge computing, TensorFlow Lite, TPU, device, model, metrics

## Abstract

In recent years, more and more devices are connected to the network, generating an overwhelming amount of data. This term that is booming today is known as the Internet of Things. In order to deal with these data close to the source, the term Edge Computing arises. The main objective is to address the limitations of cloud processing and satisfy the growing demand for applications and services that require low latency, greater efficiency and real-time response capabilities. Furthermore, it is essential to underscore the intrinsic connection between artificial intelligence and edge computing within the context of our study. This integral relationship not only addresses the challenges posed by data proliferation but also propels a transformative wave of innovation, shaping a new era of data processing capabilities at the network’s edge. Edge devices can perform real-time data analysis and make autonomous decisions without relying on constant connectivity to the cloud. This article aims at analysing and comparing Edge Computing devices when artificial intelligence algorithms are deployed on them. To this end, a detailed experiment involving various edge devices, models and metrics is conducted. In addition, we will observe how artificial intelligence accelerators such as Tensor Processing Unit behave. This analysis seeks to respond to the choice of a device that best suits the necessary AI requirements. As a summary, in general terms, the Jetson Nano provides the best performance when only CPU is used. Nevertheless the utilisation of a TPU drastically enhances the results.

## 1. Introduction

Today, we live in a constantly evolving digital age, where connectivity and data processing capacity are essential to drive innovation and progress in all areas of our society. In recent years, thanks to technological advances, most of the devices that surround us are capable of collecting information, which has led to a significant increase in the amount of data generated. In turn, these devices can send information to cloud platforms where the information from a large number of devices can be concentrated, allowing more complex operations to be carried out, such as calculations of aggregates and KPIs or training and inference of artificial intelligence models.

Given this scenario, it has become necessary to find efficient solutions to handle and process this growing amount of data. In this context, the Edge Computing (EC) paradigm arises as a response to the challenges posed. One of the main reasons is the need for ultra-low latency responses in critical environments. Sending data to the cloud could cause significant delays due to the hops between nodes required to reach a server [1].

Besides low latency, EC offers a series of notable advantages when processing and analyzing data in devices close to where it is generated. On the one hand, reducing the data volume transferred to the cloud implies a reduction in the network bandwidth and, in turn, less infrastructure needed and a saving in energy costs. On the other hand, the more data that are processed at the edge layer, the less that need to be processed in the cloud. This also means cost savings when working with a cloud service provider. Another notable advantage is the improvement in privacy and security by keeping the data in a local environment, that is, without leaving the facilities of the company or entity that generated it. The EC also allows applications and services to continue to function even in situations of disconnection from the cloud or interruptions in connectivity. In addition, data processing and analysis at the edge enable real-time communication and instant decision-making without relying on communication with the cloud. Therefore, it is an ideal technology for environments with extremely limited connectivity such as offshore. Finally, distributing processing across local devices enables greater scalability and responsiveness [2].

In the last few years, the execution of artificial intelligence models within these devices has been promoted. In this way, agility and decision-making are boosted in this computing layer. This area of the EC is referred to as Embedded AI [3]. Consequently, edge devices with artificial intelligence accelerators integrated are emerging such as Graphical Processing Units (GPUs) and Tensor Processing Units (TPU).

This research focuses on exploring and analyzing in depth the field of Embedded AI and its application. Specifically, how well the so-called *edge devices* behave, at both hardware and software levels, when running machine learning models. For this purpose, an experiment has been conducted involving the deployment of different TensorFlow image classification models in diverse EC devices. Next, significant metrics are acquired to measure the hardware and performance behavior, as well as the energy consumption. Along with this, the use of the TPU as an Artificial Intelligence (AI) accelerator is analyzed in order to observe the improvements and consumption that this entails. By following this approach, we gain the ability to effectively identify and observe inherent device limitations, which is crucial for making detailed and insightful comparisons among them. Furthermore, as part of our methodology, we have developed a set of testing tools that play a vital role in graphically processing the results we’ve obtained. These tools provide a visual representation of the data, enhancing our capacity to draw meaningful conclusions from the analyses conducted. Therefore, the information obtained throughout this research enables professionals of the sector to acquire a better understanding of the behavior of the device analyzed and, consequently, it facilitates the decision-making when selecting edge devices [4,5].

Therefore, the contributions of this research can be summarised as:

1. Provide a deep overview of the embedded AI ecosystem including the different edge paradigms, the diverse device types, the embedded AI frameworks and the most utilized adaptation techniques to make an AI model compatible with resource-limited devices.
2. Analyzing edge computing device behavior with embedded AI models.
3. State key insights to enhance the device selection for embedded AI.

The rest of the paper is organized as follows: a detailed background and the related work are, respectively, provided in Section 2 and in Section 3. Next, the experiment conducted is explained and discussed in Section 4. Finally, Section 5 exposes the conclusions and future work.

## 2. Background

In this section, the different areas of knowledge that have served as the basis and inspiration for the research are analyzed. For this reason, the following subsections identify paradigms of interest related to the edge, analyze different edge device families and present Embedded AI frameworks and model adaptation techniques for this computing layer.

### 2.1. Edge Paradigms

The term edge computing [6] arises due to the appearance of applications that require autonomy, do not tolerate latency, or require a large amount of bandwidth. During the last decade, there has been a great increase in Internet of Things (IoT) devices capable of generating data through sensors. Consequently, the processing of the data close to the source of generation can be more efficient in some scenarios instead of processing everything in the cloud by default [7].

To address these challenges edge computing paradigm aims to promote data processing and the execution of applications and services near the edge of the network (where the data are generated). Thus, greater efficiency and improvement in data processing and storage is achieved, as well as a reduction in latency and transmission [8].

**Figure 1:**
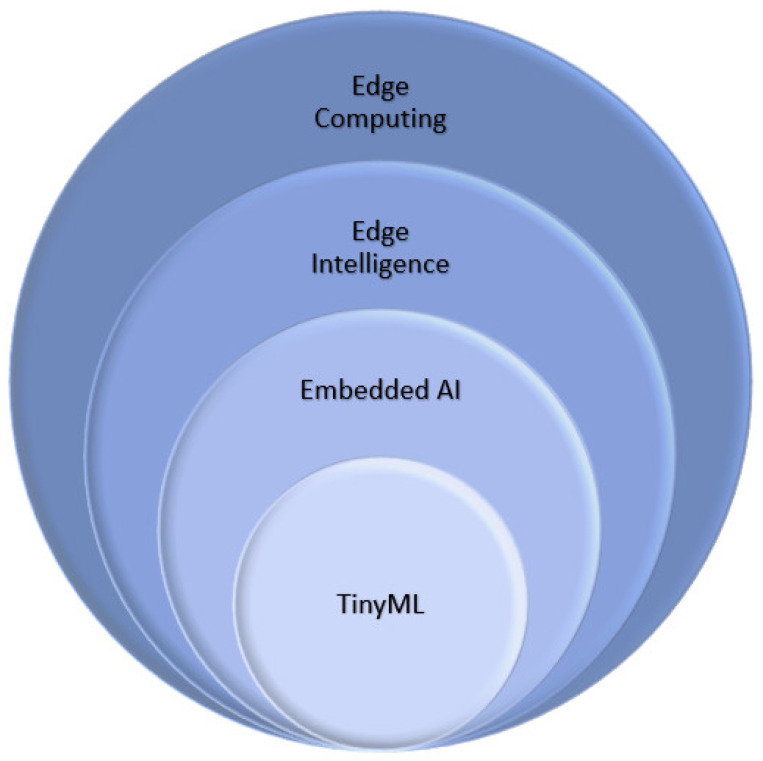
Relationship among different Edge paradigms.

The term edge intelligence [9] refers to the application of analytic techniques such as making inferences with a machine learning model in an edge device. As defined in Mwase et al. [10], the type of devices utilized in the edge layer can be very diverse ranging from resourceful hardware with higher latency to very limited hardware battery-powered devices that enable ultra-low latency. Consequently, the application of machine learning techniques in devices with reduced hardware like system on chip (SoC) is denominated embedded AI [3] and when the hardware is still more constrained like in micro-controller units (MCUs) is Tiny Machine Learning (TinyML) [11]. The relationship between these paradigms is illustrated in Figure 1.

### 2.2. Edge Device Families

There exist numerous embedded devices which can be classified in different ways, according to the function they perform, their complexity, the supported technology, etc. For this work, the focus has been put on Integrated Circuits (IC), also known as a chip or microchip. It is a small structure, normally made of silicon, on which printed electronic circuits are manufactured. They can be part of a Single-Board Computer (SBC) based on a System on Chip (SoC) or Field Programmable Gate Array (FPGA) or Microcontroller Unit (MCU) [12].

Due to the rapid evolution of technology in recent decades, it has been possible to integrate most of the functional elements of an electronic system on a single chip. SBC is a whole computer constructed on a single printed circuit board that contains memory, processor, I/O devices, and other slots. It is based on a SoC which has all the components integrated into it. A SoC is an integrated circuit that integrates a complete electronic system into it. The main function of these elements is to reduce the size of the device, decrease the cost, as well as increase efficiency and performance. The main components that include these elements are CPU, RAM memory, input and output controllers, GPU, communication controllers (Wi-Fi, Ethernet, etc.) and even in some cases TPU. In general, SoCs do not have an operating system built into the chip itself. Instead, the operating system runs on the SoC’s CPU and is loaded into memory when the device is powered on or rebooted. SoCs are used in a wide variety of applications such as phones, mobiles, tablets, IoT devices, game consoles, network equipment, etc. Examples belonging to this family are Google Coral Dev [13], Google Coral Dev Mini [14], Raspberry Pi4 [15], Nvidia Jetson Nano [16] or Hummingboard Pro [17].

An MCU [18] could be defined as an electronic device made up of a programmable integrated circuit capable of executing the logical processes recorded in its memory. The objective of these devices is to automate processes and process information. They are used in cases in which it is required to follow an automatic process depending on the information that arrives from the different input elements. The main components are: CPU, memory, main clock and input/output peripherals. Nowadays, microcontrollers are used in a vast number of products such as household appliances, toys, vehicles, hardware devices, control and measurement devices, etc.

FPGAs [19] are prefabricated silicon devices, made up of multiple complex and programmable digital circuits. They consist of a two-dimensional array of configurable blocks that can be connected to each other. Through programming, they can be converted and customized into almost any type of digital circuit or system. The main advantage of using FPGAs is their flexibility and scalability since they can be quickly adapted to changes in requirements. In addition, they are characterized by the high performance they offer, compatibility with other components and their low consumption and cost. These devices are used in a wide variety of applications like industry, research and development.

Table 1 shows the main characteristics of these edge device families to better compare them. It defines the main *purpose* to utilize a specific device family, the *CPU* processing capabilities and architecture, the information about the hardware *components* integrated, the *computing capacity* to quantify the performance, categorizing it as high, variable or low, the *energy consumption* rates energy efficiency (crucial for power-constrained devices), the *flexibility* to evaluate the adaptability from low to high, the *programmability* to identify customization facility and, finally, the financial aspect.

### 2.3. Embedded AI Frameworks

TensorFlow [20] is an open source framework developed by Google and aimed at building and training ML models and deep neural networks. Its flexible, cross-platform architecture allows it to work with CPUs, GPUs and TPUs [21]. TensorFlow Lite (TFL) [22] is an optimized version of TensorFlow for adapting machine learning models for embedded devices. In addition, it can optimise the performance, reduce the consumption and improve the latency. One of the advantages to highlight is that it provides a set of tools for optimizing models or applying quantification techniques. In addition, it offers support for hardware acceleration. In turn, TensorFlow Lite Micro (TFLM) [23] is an optimized version of TensorFlow Lite designed for very limited embedded systems, such as very simple IoT devices and microcontrollers. These devices have large power, storage and memory constraints (just a few kilobytes). That is to say, it has been conceptualized for TinyML.

PyTorch [24] is an open source framework used to develop and train machine learning models. It allows the creation of deep learning models using a simple syntax and a flexible architecture. In turn, PyTorch Mobile is an extension of PyTorch that allows running machine learning models on mobile devices and embedded systems. The main objective is to be able to run the models on devices with limited resources in an efficient and fast way [25].

Edge Impulse [26] is a development platform aimed at training and deploying machine learning models and conducting signal analysis in embedded devices. It provides a usable graphical interface enabling the building of projects without requiring programming skills.

### 2.4. Embedded AI Model Adaptation Techniques

Model adaptation techniques for Embedded AI are a set of strategies and tools that allow adapting AI models in embedded systems with limited resources. These techniques focus on optimizing and reducing the size of the model. Consequently, it can run on more reduced hardware requiring less amount of resources, while maintaining acceptable accuracy.

The most used techniques are **Weight Pruning** which consists of the elimination of the weights that contribute least to the precision of the models. To this end, the weights with values close to zero or having a small influence are removed. This technique can be applied to different types of AI models, such as convolutional neural networks (CNN) or recurrent neural networks (RNN). In addition, it can also help to avoid overfitting in AI models, since reducing the number of parameters reduces the model’s ability to memorize data instead of learning underlying patterns [27,28]. **Compression** technique is based on the combination of adjacent layers in a single layer. Thus, the total number of layers is reduced and, as a consequence, the model size [29]. The main goal of the **quantization** technique is to reduce the precision of model parameter values from high-precision floating-point numbers to low-precision integers. This is achieved by assigning a limited range of discrete values to each parameter, which reduces the number of possible values they can take. In most cases, AI models are trained with high precision and quantized after training. Quantization can be applied at different stages of the model. It is also possible to use different quantization strategies, such as uniform quantization or data-aware quantization [30,31,32].

## 3. Related Work

In this section, relevant literature related to this research is reviewed. In addition, specific research efforts that experiment with the behavior of machine learning models when deployed on edge devices are examined and compared with this analysis. For this purpose, the main aspects of this study are listed below, and next, Table 2 contrasts them with the reviewed papers that offer experiments:

- Measurement of different metrics: inference time, consumption of energy, RAM and CPU. In addition, the inference time of TPU devices is measured in both modes: with the TPU disabled and enabled. Thus, it can clearly examine the advantages of using such an AI accelerator.
- Focus on different machine learning models, instead of just on a single model.
- Utilisation of diverse edge devices: Raspberry Pi 4, Google Dev coral and Coral Mini, Nvidia Jetson Nano and HummingBoard Pro.

A systematic survey of AI accelerators for edge environments is presented in [33]. This article examines both hardware and software criteria and evaluates representative AI accelerator products. Additionally, it summarizes current trends and future directions in AI accelerator design. This work is valuable for deeply understanding the edge AI accelerators alternative, as well as to better learn the state of the art in this area. Sipola et al. [34] provide a review that analyzes the development of Edge AI applications and offers a perspective of both the hardware and software alternatives utilized in this area. To this aim, it discusses hardware products in various categories and also focuses on emerging trends in Edge AI software, including neural network optimization and software development for mobile devices and microcontrollers. Imran et al. [35] review the development boards available for running AI algorithms at the edge, providing valuable insights into the options available in terms of hardware for Edge AI applications. Merenda et al. [36] provide a detailed review of models, architectures and requirements for implementing machine learning in Internet of Things (IoT) devices. Of particular importance is his exploration of the implementation of machine learning on microcontrollers, using a number detection model. This review sheds light on the practical requirements of deploying deep learning on resource-constrained devices, which is consistent with our focus on edge computing devices. These studies collectively contribute to the understanding of edge computing device capabilities, performance metrics, and practical considerations for deploying deep learning algorithms at the edge, providing valuable context for our research on edge-device analysis.

Regarding the experiments evaluated and contrasted with our research, in DeepEdgeBench [37], authors present a comprehensive analysis comparing the performance in terms of inference time and power consumption of various edge devices, including Asus Tinker Edge R, Raspberry Pi 4, Google Coral Dev Board, Nvidia Jetson Nano and Arduino Nano 33 BLE.

Hadidi et al. [38] characterize various commercial edge devices using popular frameworks employing well-known convolutional neural networks (CNNs). The research analyzes the impact of implemented frameworks, software stacks, and optimizations on device performance. Additionally, they measure power consumption and temperature behavior, offering insights into the limitations and capabilities of these edge devices. Conversely, this study considers also the analysis of the underlying hardware behavior.

**Table 2:** Comparative between different experiments.

ASPECTS	DeepEdge Bench [37]	Hadidi et al. [38]	EdgeFaaS Bench [39]	Yolo Benchmark [40]	Kang et al. [41]	DL Bench [42]	Antonini et al. [43]
Measure inference/ execution time	✓	✓	✓	✓	✓	✓	✓
Measure inference time in TPU- devices both enabling and disabling the AI accelerator	✓	-	✓	-	-	✓	-
Measure energy consumption	✓	✓	-	✓	✓	-	✓
Measure RAM memory consumption	-	-	✓	✓	✓	-	✓
Measure CPU consumption	-	-	✓	-	-	-	-
Test with different models	-	✓	✓	-	✓	✓	✓
Test with Raspberry PI4	✓	-	✓	✓	-	-	✓
Test with Google Coral Dev Mini	-	-	-	-	-	-	-
Test with Google Coral	✓	-	-	-	✓	-	✓
Test with Nvidia Jetson Nano	✓	✓	✓	✓	✓	✓	✓
Test with HummingBoard Pro	-	-	-	-	-	-	-

EdgeFaaSBench [39] is a benchmark suite aimed at testing edge devices under a serverless computing paradigm. EdgeFaaSBench includes 14 different benchmark applications and measures various system- and application-level metrics, including system utilization, application response time, hot/cold start time, and the impact of attendance. Experiments are performed on widely used edge devices to demonstrate the ability of EdgeFaaSBench to collect various metrics to understand the effectiveness of serverless computing at the edge. Nonetheless, this benchmark does not offer the possibility to measure the energy consumption of the underlying devices. In addition, currently, it only supports Raspberry Pi4 or Jetson Nano as edge devices and, consequently, the analysis of TPU usage is not considered.

Yolo benchmark [40] investigates the inference workflow and performance of the YOLO object detection model on three accelerator-based edge devices, including NVIDIA Jetson Nano, NVIDIA Jetson Xavier NX and Raspberry Pi 4B (RPi) with Intel Neural Compute Stick2 (NCS2). Different video contents with different input sizes are compared using four different versions of the YOLO model on such three devices. This research is focused on the evaluation of the Yolo model (including different versions) in different edge devices. Contrarily, this paper is focused on the behavior of edge devices and, hence, different algorithms are selected to avoid these biases.

Kang et al. [41] evaluate a set of AI models in two edge devices powered by AI accelerators: Nvidia Jetson Nano and Google Coral Dev. Authors follow a device ad-hoc specific process to load the models. Conversely, the experiment of this paper standardizes such a process and, hence, the comparison among devices is more realistic. Moreover, they consider only two different devices, instead of five.

The DL models benchmark [42] focuses on studying how the Edge devices NXP i-MX8M-PLUS and Jetson Nano behave in five different object detection DL models. This article focuses more on the models themselves, as it describes the process of converting the models to the format needed for both devices. Furthermore, the only parameter it measures is time.

Antonini et al. [43] performs a benchmark on seven different platform configurations, each equipped with one of three edge accelerators: Google Coral, NVidia Jetson Nano, and Intel Neural Compute Stick. The analysis focuses on latency, memory usage, and power consumption, and provides empirical insights into how these accelerators specifically handle deep learning tasks at the edge.

As Table 2 highlights, while all of them measure execution or inference time and most also measure power consumption, only one of them measures CPU usage. In addition, this one has only used two devices on which this metric has been analyzed and none of them are TPU-based. Three of the works use TPU-accelerated devices such as Google Coral. On the other hand, devices such as Google coral mini and HummingBoard Pro are not considered in any of these studies. Consequently, there is a gap in conducting this study as TPU is considered more specifically. On the other hand, in this experiment, we have also compared how the devices behave when they use the AI accelerator and also when they do not. This has only been conducted in three of the articles analyzed and not all have used the same devices. It should also be noted that this article has been the only one that has measured the RAM memory consumption of Coral devices when the AI accelerator (TPU) is used and not. Thanks to this, it has been possible to study how the use of TPU influences RAM consumption. Furthermore, with the rapid advancement of cutting-edge devices and artificial intelligence models, we strongly believe that these types of research experiments should be conducted periodically to update this fast-moving state of the art.

## 4. Experiment

The objective of this experiment consists of evaluating a set of market-used systems on chip edge devices. Specifically, how they behave when executing machine learning models. In addition, some of these devices are equipped with AI accelerators and it is relevant to understand the difference when they are utilized or not. For this purpose, a set of key metrics are considered to better understand the device behavior, like CPU and RAM percentage, inference time or energy consumption.

This section details the experiment carried out in this research. Analysis and design are detailed in Section 4.1. In addition, this subsection also integrates preparation aspects to simplify reading the article and because some of its contents exceed its scope. Subsequently, the Section 4.2 explains the execution and the Section 4.3 discusses the results of the experiment. A global discussion is made in the Section 4.4. Finally, in Section 4.5 the results obtained in the research studied in Section 3 are analyzed.

### 4.1. Analysis, Design and Preparation

In order to better explain the process of analysis, design and preparation, this section is structured as Section 4.1.1 and examines the devices to select for the experiment, in Section 4.1.2 the models utilised are detailed, Section 4.1.3 defines the metrics to consider, the data treatment is explained in Section 4.1.4 and, finally, in Section 4.1.5 the software requirements.

#### 4.1.1. Devices

The device selection is the first step to carry out since the purpose is to analyze specific devices for embedding AI. For this experiment, only devices from the same family are utilized to compare devices with similar characteristics, in order to make it as real and accurate as possible. Specifically, devices from the SoC family are utilized. This is due to the fact that by comparing the devices at the hardware and software level, the big differences between SoC and MCU-type devices can easily be observed. For example, RAM memory is critical when working with artificial intelligence models since it is required to load the model, its weights, input data, etc. In SoC devices, it ranges from 1 to 8 GB, while for MCUs the maximum value is 4MB which is a significant difference. Despite existing many approaches to embed AI in MCUs (the TinyML area), this research is focused on SoC because of their power to integrate AI models with no or few adaptations from the original model.

Therefore, the following five market-representative edge devices have been selected: Google Coral Dev Board, Google Coral Dev Board Mini, Jetson Nano, HummingBoard Pro and Raspberry Pi 4 Model B. Their main features can be observed in Table 3. The characteristics analyzed in this table are the following: the *CPU* of the device, the *TPU* support, what *Clock frequency* it has, the *GPU* integrated and the *RAM* size and its model, the *external memory* support and the market *approximate price*.

In order to perform the experiment on the devices, they must first be booted and properly configured. Then, the Python modules defined in Section 4.1.5, the models detailed in Section 4.1.2 and the input images and labels are copied to each device. The module in charge of loading the models can easily be configured to enable the TPU AI accelerator or not. Consequently, each experiment targeted for both Google Corals is executed twice as if two different devices would be. However, TensorFlow Lite can not make use of the GPU in Python applications [44] and we did not want to use it for some experiments in TensorFlow models and for other TensorFlow Lite ones. Consequently, unfortunately, Jetson Nano only makes use of its CPU in this experiment. In future work, we will explore the utilization of the GPU.

Afterward, the libraries and dependencies that must be installed on the devices are: TFlite to run TensorFlow machine learning models on devices with limited resources. Pillow provides functions to open, manipulate, and save images in Python and Psutil to obtain information about the operating system and the use of system resources, such as CPU or memory.

On the other hand, it is important to note that a separate hardware component has been utilized to measure the energy consumption on the devices, the Intertek JGQ02S-01 [45] energy meter model. This device is specifically designed to accurately measure the power consumption of a device and, hence, provide the necessary information for our study.

#### 4.1.2. Models

For the model provision, the TensorFlow framework was chosen since it facilitates the creation of models adapted for this type of device by using TensorFlow Lite. Indeed, two of them were directly downloaded in TFL-compliant format and the other one was converted to it.

The three models belong to the deep learning image classification field to subject the devices to similar conditions during the three experiments. This way, if a model produces anomalous results, the risk of experiment contamination is balanced by having selected similar models.

The first time a model is executed, it is loaded in memory, which implies a longer execution time. Conversely, the execution time is reduced in the subsequent inferences since the model is already loaded in memory. Therefore, each model is executed several consecutive times in a loop to clearly identify how much time the model execution takes in both circumstances.

In Table 4 we see the main features of the chosen image classification deep learning models:

#### 4.1.3. Metrics

In order to better investigate the behavior of the edge devices the following metrics are registered during the experiment execution.

The CPU and RAM are measured to evaluate the efficiency of the devices when executing the model and analyze their degree of saturation. RAM is mainly used to load the model (including its weights) and the images to be classified. Five minutes before and after running the model are also recorded to allow comparison of a device’s hardware resources when running a model or at rest. To obtain these metrics, the mathematical Formula (1) for the CPU percentage and the mathematical Formula (2) for the RAM percentage have been used.

(1) $$\mathrm{CPU}\;\mathrm{percentage}=\frac{\mathrm{CPU}\;\mathrm{usage}\;\mathrm{capacity}\;\mathrm{at}\;a\;\mathrm{specific}\;\mathrm{time}}{\mathrm{Total}\;\mathrm{CPU}\;\mathrm{capacity}}\times 100$$

(2) $$\mathrm{RAM}\;\mathrm{percentage}=\frac{\mathrm{RAM}\;\mathrm{usage}\;\mathrm{at}\;a\;\mathrm{specific}\;\mathrm{time}}{\mathrm{Total}\;\mathrm{RAM}}\times 100$$

Inference times are registered to assess how fast a device is for executing models. This metric is very valuable to identify if the performance increases when using TPUs. Furthermore, energy consumption is taken into account to enable trade-offs between the power of a device and the energy required to make it work or facilitate decisions such as enabling or not TPU accelerators.

Unfortunately, it is important to note that these devices do not have a separate Graphics Processing Unit (GPU) or Tensor Processing Unit (TPU) since it is integrated with the Central Processing Unit (CPU). As a result, this makes it impossible to measure the GPU or TPU utilization and, hence, regarding processors only the measurement of the CPU utilization is considered.

#### 4.1.4. Data Treatment

The data registered (in JSON format) are manually extracted from the devices at the end of each experiment. This way, it is not required to be programmatically sent over the network which would significantly increase the hardware measures. Subsequently, these data are processed by a Python 3.10 application (see Section 4.1.5) and stored into ElasticSearch [48] (version 7.13.4) which enables the creation of a Kibana [49] graphical dashboard to boost data comparison and analysis.

This dashboard provides a dedicated graph for each metric defined in Section 4.1.3. Moreover, a select box element enables the visualization of the data separated by the model used. Finally, a table is also integrated to consult the original data, including the output of the inference, and to facilitate data filtering. As a result, the behavior of a model on different devices can be better analyzed.

#### 4.1.5. Software Requirements

On the one hand, several Python modules have been designed to carry out the following experiment features. For this purpose, Python 3.10 version has also been used:

- **Model manager module** for loading the different TensorFlow Lite deep learning classification models in a standard fashion. This way, all the models are loaded in all the devices using the same piece of code and, consequently, avoiding the use of device-specific wrappers which could modify the experiment results. In addition, this code will also be in charge of recording the inference time of each input and the total experiment execution time.
- **Metrics manager module** for capturing every 0.2 s the hardware resource metrics both when executing models and at rest (before and after such model executions). The results will be saved in a JSON file that will be used later to obtain the relevant conclusions.
- **Data manager module** for automatically processing the data registered both from hardware resource metrics and time metrics. In addition, this module is responsible for creating the relevant ElasticSearch indexes, storing the data and generating statistical values for each metric (average, maximum, minimum and standard deviation). This automation task allows us to always process the data in the same way regardless of the model or the device. This way, new models and devices could quickly be integrated as an extension of the experiment.

On the other hand, Docker and Docker Compose have been used to quickly make ElasticSearch and Kibana available for this research context.

**Figure 2:**
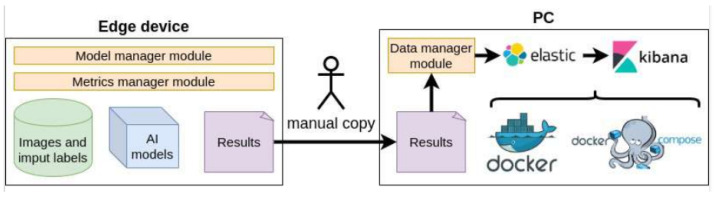
Experiment software requirements.

Figure 2 shows a global vision of the software components involved in the experiment. Each device is prepared with the Model and Metrics manager modules (and their required libraries), the image classification models and the input images and labels. The results obtained from executing the experiments are manually copied to the laptop where the data are analyzed. On the laptop, ElasticSearch and Kibana containers are available by means of using Docker Compose. Next, the necessary Kibana dashboards can be also prepared. Finally, the Data manager module can be executed to treat the data, create the corresponding ElasticSearch indices and store the data on them. At the end of this process, metric dashboards are populated with the experiment data to clearly and quickly analyze the behavior of the devices when executing the models.

### 4.2. Execution

After the definition, design and preparation phases, the experiment execution phase is conducted for the three models in the five devices. For this purpose, an additional simple Python module orchestrates the execution of the Model and Metric manager modules on each device for each model. Thus, both modules are synchronized to manage the measures acquisition when the models are being executed and the required five minutes before and after.

Regarding the hardware, the energy consumption device is plugged in during each experiment to take the corresponding measures. Once all the experiments have been carried out, the data are manually extracted from the devices to a laptop and the Data manager module is executed to treat the data, create the ElasticSearch indices and upload the data to ElasticSearch to feed the Kibana dashboard.

### 4.3. Results and Discussion

First of all, it is worth mentioning that the experiments have been successfully carried out on all the devices, except for HummingBoard Pro. After booting it and installing all the necessary libraries, the execution of the classification process returned an error with the text: *Illegal instruction*. Apparently, the cause of this error is that the processor (NXP i.MX 6) of the HummingBoard Pro does not support TFL. The documentation does not explicitly state it but, conversely, the documentation for the HummingBoard Ripple device (i.MX8M processor) clearly states its compatibility with TFL.

Another aspect to mention is that the results obtained with the different models are equivalent. That is to say, all the machines behave similarly independently of the deployed model.

The rest section discusses the results obtained through the execution of the experiment after having analyzed the different proposed metrics. To achieve this, we have structured the analysis into subsections associated with the diverse metrics studied. Section 4.3.1 delves into CPU usage, in Section 4.3.2 addresses the RAM usage in percentage and the absolute RAM consumption, in Section 4.3.3 inference and execution times are studied; next, Section 4.3.4 analyses the energy consumption and, once all the metrics have been studied Section 4.4 provides a global discussion. It is worth noting that the graphs presented correspond to a specific model but the behavior resulted to be similar for all of them.

#### 4.3.1. Cpu Percentage

**Figure 3:**
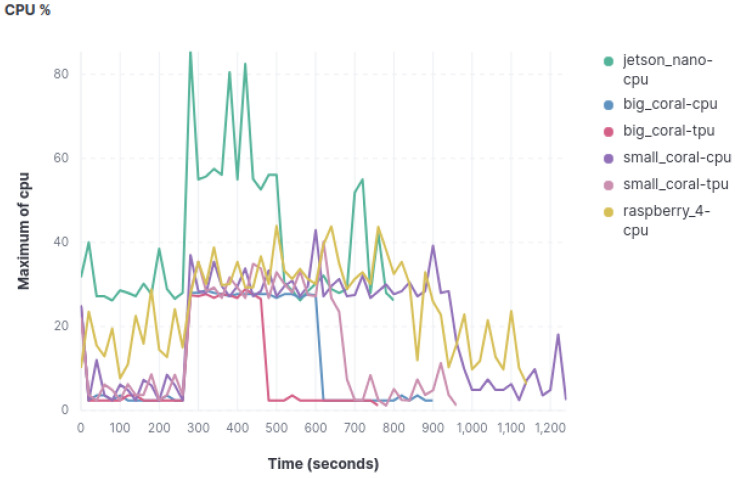
Graphics with CPU usage.

Figure 3 shows the graph with the results of the CPU usage in the object classification model. Results for the other two models are equivalent. Notably, the Jetson Nano stands out as the highest CPU consumer, both during the execution of the AI models and when only acquiring metrics. The Raspberry Pi, on the other hand, takes the second spot in CPU usage, with occasional minor peaks during the experiment that do not reach the Jetson Nano’s minimum CPU utilization levels. Additionally, the Raspberry Pi exhibits no significant CPU spikes during image classification.

In contrast, the two Coral devices exhibit highly similar behavior across different models. It is worth emphasizing that CPU usage in both Coral devices remains nearly identical, whether utilizing the TPU or not during image classification.

#### 4.3.2. Ram Percentage and Total

For the analysis of RAM consumption, two approaches have been employed. Firstly, the percentage of RAM consumed by each device was assessed, in order to see the saturation to which the RAM was subjected during the experiment. Secondly, since not all devices have the same amount of RAM, the total amount of RAM consumed was also analyzed.

**Figure 4:**
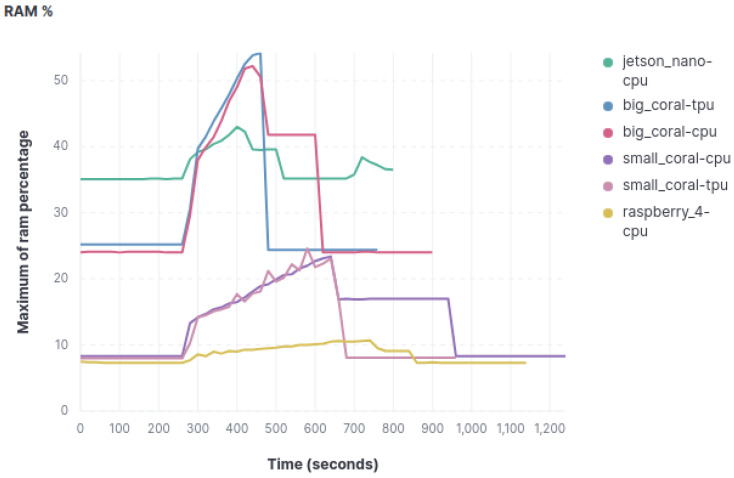
Graphics with RAM percentage usage.

The analysis of RAM usage percentage, according to the data in Figure 4, which represents the object classification model, yields the following conclusions. Undoubtedly, the Raspberry Pi stands out as the device that utilizes the least RAM percentage among the three models but this is also because it is the one with more RAM capacity. The Coral Mini device exhibits RAM consumption patterns similar to the Raspberry Pi, although more frequent spikes in RAM usage are observed during the image classification process.

The next device that records a higher RAM usage percentage is the big Coral device, consuming just over twice as much compared to the Coral Mini. Furthermore, the spikes in RAM usage during classification are more pronounced. Similar to CPU usage, minimal differences in resource consumption metrics are observed when both Coral devices use or do not use the TPU.

Finally, the Jetson Nano proves to be the device that consumes the most RAM resources in normal operating conditions. Nevertheless, during image classification, it experiences fewer significant spikes, occasionally being surpassed by the big Coral device in some cases.

**Figure 5:**
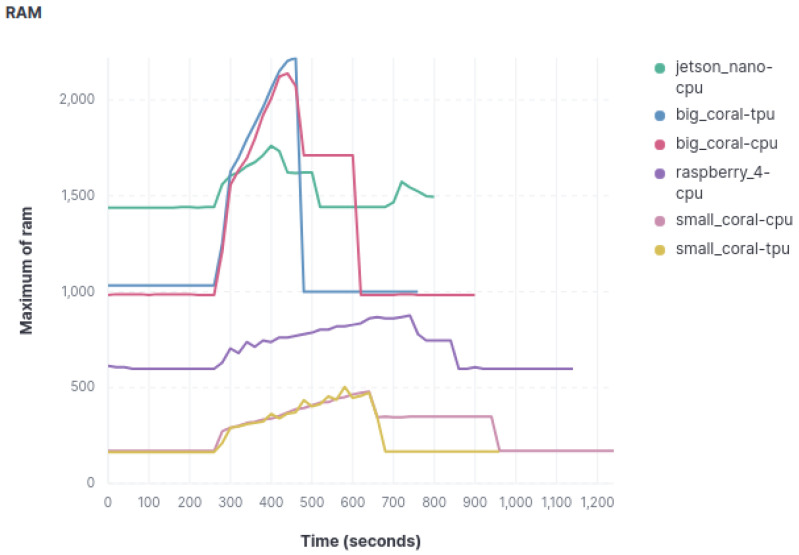
Graphics with RAM usage.

As we mentioned before, not all devices have the same amount of RAM, so we have also recorded the RAM total consumption, as can be seen in Figure 5, which shows the results for the object classification model. The conclusions regarding absolute RAM consumption follow a similar trend to those obtained when assessing the percentage of use. However, it is noteworthy that, in this case, the Coral Mini emerges as the device with the lowest RAM consumption, reversing its position in the previous ranking with respect to the Raspberry. The remaining observations and conclusions concerning the other devices remain consistent.

#### 4.3.3. Inference Time

**Figure 6:**
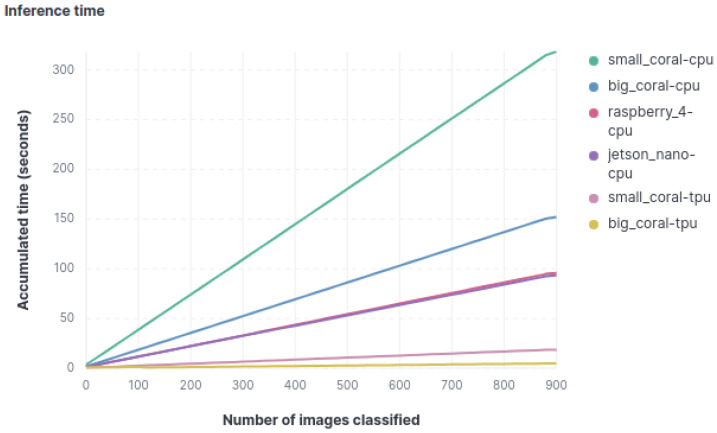
Graph with accumulated classification time.

The cumulative inference time for each image has also been measured to identify how much time each device takes to classify the entire set of images for each model. Figure 6 displays the total time required by each device to complete the classification task in the object classification model. Unlike other metrics, there is a significant difference between both Coral devices when using the TPU and when not using it. The difference is so pronounced that when using the TPU for classification, the Coral Mini becomes the second-fastest device, being surpassed only by the big Coral. Without TPU utilization, the device order, from fastest to slowest, is as follows: Jetson Nano, Raspberry, big Coral, and Coral Mini.

#### 4.3.4. Energy Consumption

Table 5 presents the average values of the energy consumption of each device (considering the three models) and the maximum values on each of the three experiments (birds/objects/numbers). As previously mentioned, the power consumption of the devices has been assessed in the different experiments using a power meter plug. When no classification task is running, it is observed that the most energy-efficient device is the Coral Mini. In contrast, both the Jetson Nano and the Raspberry Pi consume approximately twice as much power as the Coral Mini, and their power consumption levels are quite similar to each other. On the other hand, it is worth noting that the big Coral is the device with the highest power consumption.

Another significant conclusion is that in both Coral devices, power consumption remains at similar levels, whether the TPU is in use or not. Therefore, surprisingly, we can conclude that the use of the TPU does not affect energy consumption. Additionally, concerning the recorded peaks of maximum power consumption, it is observed that all devices experience an increase of around 2 watts. This increase is particularly noteworthy in devices that initially consume less power, as it represents a higher percentage increase in their total power consumption.

### 4.4. Global Discussion

From the results obtained and from the metrics studied, a general analysis of the devices can be conducted. The following conclusions can be highlighted:

1. the devices that achieved the best metrics are the Google Coral Dev Board and the Google Coral Dev Board Mini when utilizing the TPU. These devices are the fastest in terms of classification time and, in general, do not require very powerful hardware. Furthermore, their price is relatively affordable compared to other analyzed devices. Between the two, the Google Coral Dev Board yields slightly superior results, although it is slightly more expensive than the Google Coral Dev Board Mini. However, the performance of both devices significantly decreases when the TPU is not used.
2. the Jetson Nano is the device that obtains the best results when exclusively using the CPU. Despite not employing an AI accelerator, it demonstrates good performance and offers solid features. Although its price is somewhat higher and it consumes more resources under normal conditions, it exhibits a lower number of performance spikes during image classification, indicating greater resilience.
3. the Raspberry Pi stands out for its low resource consumption in most of the measured hardware components. However, during image classification, it shows higher resource consumption and is the slowest device in terms of speed. Its primary advantage lies in its affordable price.
4. We have not been able to take advantage of all the virtues of the Jetson Nano because of the TFL limitation with GPU Python delegate but at least the experiments have been conducted successfully. Conversely, it was an error to include the HummingBoard Pro in the experiment due to its incompatibility with TFL models.
5. All the edge devices behave similarly to the different models targeted to deal with image classification problems.

It is important to note that this analysis is specific to the designed use case, and it is expected that these devices will behave similarly in other environments. Additionally, consumer choices may depend on their priorities, whether it be speed, price, or other factors. However, due to the fact that while using the TPU the energy consumption, RAM and CPU are not affected, we highly recommend its utilization when applicable because of its satisfactory results.

### 4.5. Findings in Related Works

This section analyses the results of similar works. Similarly to this study DeepEdgeBench [37] states that for a Tensorflow model that can be quantized and converted to TFLite format, the Google Dev Coral device delivers the best performance, both for inference time and power consumption. In Hadidi et al. [38], in most cases, either GPU-based devices or EdgeTPU provides the best performance. However, this research is not fully comparable since they test the Jetson Nano with the GPU. EdgeFaaSBench [39] concludes that the inference times for the Jetson Nano CPU have been better than those of the Raspberry Pi. Additionally, the inference times for the Jetson Nano GPU are much better than the Raspberry Pi CPU and the Jetson Nano CPU. Yolo benchmark [40] after performing the experiment, it concludes that the inference performance of an accelerator-based SBC depends mainly on the AI model. Kang et al. [41] deduces that Coral Dev Board shows about 5 times better performance than the Jetson Nano for relatively simple CNNs with a small number of parameters. Additionally, Coral Dev Board uses an order of magnitude less memory than Jetson Nano in all tests. The DL models benchmark [42] demonstrates that the i-MX8M-PLUS device performed slightly better in general. The performance improvement of co-processor models compared with CPU models is about 10 times in the i-MX8M-PLUS and 5 or even worse in the EdgeTPU. Antonini et al. [43] has detected some interesting findings such as that the execution time is very different, depending on the edge platform. Overall, the Coral Dev and Coral (RPi 4B) outperform the other devices.

Regarding energy consumption, DeepEdgeBench [37] experiment says that when using just CPU, the Jetson Nano consumes the least amount of power for all models, followed by the Raspberry PI. However, the device that consumes the least energy using dedicated AI is the Coral Dev Board. Hadidi et al. [38] analyzes the results obtained and concludes that the device that consumes most is the Raspberry Pi and the one that EdgeTPU less, among both is the Nano Jetson. Yolo benchmark [40] has come to the conclusion that in terms of RPi + NCS2, it always has lower average power, no matter which model is running for inference, but its FPS is higher than Jetson Nano, which means it has better energy consumption performance than Jetson Nano. Kang et al. [41] has determined that during the benchmark run, the Coral Dev Board used 5.5 watts on average, which is 10 percent less than the Jetson Nano. However, the Coral Dev Board was measured to use 4.8 watts in idle time, which is more than double the power consumed by the Jetson Nano. Antonini et al. [43] has found that stand-alone devices (i.e., Coral Dev and Jetson Nano) consume less power when idle compared to accelerators that require a host device to operate (i.e., NCS2 and Coral Accelerator).

Concerning RAM memory consumption, EdgeFaaSBench [39] has experienced that, in general, applications on Jetson Nano use more memory than applications on Raspberry Pi 4B, and in some cases it is also significantly higher. Additionally, the Jetson Nano uses a lot of memory in GPU-enabled configurations. Yolo benchmark [40] concludes that the memory usage of GPU-based SBC (Jetson Nano or Jetson Xavier NX) is much bigger than the memory usage of Raspberry Pi + NCS2 when using the same AI model. After carrying out the experiments, Kang et al. [41] has shown how the Jetson Nano consumes 30 percent of RAM, while the Google Coral Dev Board only 5 percent. Antonini et al. [43] has noticed that the Jetson Nano device is allocated significantly more memory for GPU runtime.

Finally, the conclusions of the only work that takes CPU consumption into account are analyzed. EdgeFaaSBench [39] has managed to demonstrate that the Jetson Nano when using the GPU requires approximately the same CPU usage as the Raspberry Pi. However, when the Jetson Nano only uses the CPU, it consumes more than twice as much as the Raspberry Pi.

In this research, we have also demonstrated that Google Coral Mini, which is not considered in other studies, is in the second position after the other Coral. Furthermore, it has been possible to study how the use or not of TPU in both Corals affects the RAM energy consumption in each case. Another point in which this research differs from the rest is in the behavior of the CPU when the TPU is used and when not in the two Coral devices. Finally, another different insight reached in this experiment is that the utilization of the TPU does not affect energy consumption since it remains similar to when TPUs are not used.

## 5. Conclusions and Future Work

This article has focused on acquiring a deeper understanding of edge computing devices, specifically within the area of Embedded AI. For this purpose, a complete background is detailed in Section 2, where, initially, a comprehensive study of key concepts and paradigms in this domain was conducted to establish a robust context. Subsequently, various EC device families were explored in detail, with a specific emphasis on SoC, enabling a full immersion in this field and significantly expediting the learning process. Next, the most common embedded AI frameworks are described and, at the end of this section the most used model adaptation techniques for edge devices are cited. With the elaboration of this background, we can determine that a robust baseline in the field of embedded AI has been established by approaching the area from diverse necessary perspectives.

Subsequently, the foundations were laid to carry out an experiment aimed at collecting valuable information to select suitable devices for the deployment of Artificial Intelligence models. Prior to the design of the experiment, a thorough analysis of previous related work was carried out to underline the distinctive approach, as detailed in Section 3. Next, a meticulous definition and design of the experiments followed, which involved the selection and analysis of essential components: devices, models, metrics, data management and required code. After this, the experiment preparation was carried out, the machines were configured and adjusted, and three Artificial Intelligence models in TFL format were deployed for image classification. Additionally, two Python modules were installed in the machines: one to load and run different TFL models while collecting the inference time and the other to monitor the hardware resources of the EC devices. Then, the experiment is executed and the results are processed with an additional Python module which also is in charge of creating ElasticSearch indexes and populate them to fed a Kibana Dashboard with the data. Finally, analysis and discussion of results were conducted, offering a detailed breakdown of each metric and a global perspective.

Besides providing a solid context and a software ecosystem to benchmark edge devices for embedded AI, the following specific conclusions have been reached after the experiment analysis:

1. The experiment has determined that the energy consumption, the RAM and the CPU are not significantly varied when using or not the TPU accelerator.
2. The utilization of the TPU dramatically reduces the inference time
3. All the devices analyzed except the Humming Board Pro can be utilized to deploy embedded AI based on TFL.
4. At least for the image classification area, the devices behave similarly to different models.

Taking these conclusions into account, we must also highlight the effort made to distance ourselves from the different research analyzed, in order to be able to contribute new ideas and focus in this area.

As a general summary, we can conclude that this research work offers a significant contribution to help professionals in the decision of selecting the most adequate edge computing device for embedding artificial intelligence algorithms.

The future work will address two different directions. On the one hand, the extension of this study to better evaluate GPU-based devices like the Jetson Nano. On the other hand, the creation of a modular benchmark application enables users to assess and compare the performance of EC devices in a more personalized and precise manner. To this end, a flexible mechanism to integrate new metrics and support for additional devices will be designed.

## Figures and Tables

**Table 1:** Comparison between device families.

Characteristics	SBC (SoC)	MCU	FPGA
Purpose	General purpose or more complex applications.	Low consumption applications.	Signal processing and general purpose logic.
CPU	Powerful CPU and usually x86 or ARM architecture.	Simple CPU with low power and performance.	No integrated CPU, uses gate arrays.
Components	RAM, SO, peripherals, etc.	RAM and peripherals.	Configurable gate matrix for hardware level design.
Computing capacity	High computing capacity, graphic processing and memory.	Limited computing capacity and memory.	Computational capacity and logic are highly configurable.
Energy consumption	Higher power consumption.	Lower power consumption.	Variable power consumption.
Flexibility	Less flex.	Less flexibility, focused on specific applications.	Highly flexible and configurable.
Programmability	Fully programmable, various OS and languages.	Programmable but limited languages.	Highly programmable.
Cost	Moderate-high cost.	Low cost.	High cost.

**Table 3:** Comparison between devices.

	Raspberry Pi 4 Model B	Google Coral Dev Board	Google Coral Dev Board Mini	Humming-Board Pro	Jetson Nano
CPU	Broadcom BCM2711 with four CortexA72 cores ARM 64 bits	NXP i.MX 8M SOC (CortexA53 quad-core, CortexM4F) ARM 64 bits	MediaTek 8167s SoC (Cortex-A35 quad-core) ARM 64 bits	NXP i.MX6 Cortex- A9 from 1 to 4 cores ARM 32 bits	Four CortexA57 MPCore cores ARM 64 bits
TPU	No	ML Google Edge TPU accelerator.	ML Google Edge TPU accelerator.	No	No
Clock frequency	1.5 GHz	1.5 GHz	1.5 GHz	1 GHz	1.43 GHz
GPU	Broadcom VideoCore VI	Integrated GC7000 Lite Graphics	IMG PowerVR GE8300	Vivante GC880/ GC2000	128-core NVIDIA Maxwell
RAM	LPDDR4 2400 SDRAM 8 GB	4 GB LPDDR4	2 GB LPDDR3	DDR3 2 GB	4 GB LPDDR4
External memory	MicroSD card port	8 GB eMMC Flash	Kingston eMMC 8 GB Flash	8 GB eMMC Flash	MicroSD card port
Approximate price	115 €	160 €	100 €	280 €	220 €
URL	https://www.raspberrypi.com/products/raspberry-pi-4-model-b/ accessed 6 September 2023	https://coral.ai/products/dev-board/ accessed 7 September 2023	https://coral.ai/products/dev-board-mini/ accessed 6 September 2023	https://developer.solid-run.com/knowledge-base/hummingboard-base-pro-getting-started/ accessed 8 September 2023	https://www.nvidia.com/es-es/autonomous-machines/embedded-systems/jetson-nano/ accessed 7 September 2023

**Table 4:** Main features of classification models.

	Number Classifier	Bird Classifier	Object Classifier
Description	Load an image with a number between 0–9 and detect the number.	Classify birds appearing in an image.	Detects different types of objects.
Input data	10,000 images with digits.	100 images of different types of birds.	900 images of different kinds of objects.
Labels	No.	List of 965 labels with the types that can classify.	List of 1001 labels with the names of objects that can classify.
Framework	TensorFlow converted to TensorFlow Lite.	TensorFlow Lite [46].	TensorFlow Lite [47].
Number of layers	15	173	90

**Table 5:** Energy consumption.

	Standard (W)	Maximum (W) Birds/Objects/Numbers
Google Coral Dev Board Mini TPU	0.9–1.3	1.9/2.7/2.3
Google Coral Dev Board Mini CPU	0.9–1.3	2.2/2.6/2.1
Google Coral Dev Board TPU	4.2–4.4	5.4/6.7/5.5
Google Coral Dev Board CPU	4.2–4.4	5.3/6.4/5.3
Jetson Nano CPU	2.8–3.4	4.9/5.6/4.7
Raspberry Pi 4 Model B CPU	2.6–2.9	4.3/4.5/4.8

## Data Availability

Data are contained within the article.

## Abbreviations

The following abbreviations are used in this manuscript:

CNN	Convolutional Neural Networks
EC	Edge Computing
GPU	Graphical Processing Units
IoT	Internet of Things
MCU	Micro-Controller Unit
TPU	Tensor Processing Unit
RNN	Recurrent Neural Networks
SBC	Single Board Computer
FPGA	Field-Programmable Gate Array
TF	TensorFlow
TFL	TensorFlow Lite
TFLM	TensorFlow Lite Micro
AI	Artificial Intelligence
IC	Integrated Circuits
ML	Machine Learning
TinyML	Tiny Machine Learning
CPU	Central Processing Unit
RAM	Random Access Memory
JSON	JavaScript Object Notation
SoC	System on chip
